# Genomic epidemiology reveals statewide dispersal of clinical Shiga toxin-producing *Escherichia coli* and their antimicrobial resistome

**DOI:** 10.1128/spectrum.00956-25

**Published:** 2025-11-14

**Authors:** Ana Beatriz Garcez Buiatte, Maitiú Marmion, Samara T. Choudhury, Letícia Roberta Martins Costa, Odion O. Ikhimiukor, Samantha E. Wirth, Kimberlee A. Musser, Lisa A. Mingle, Cheryl P. Andam

**Affiliations:** 1 Department of Biological Sciences, University at Albany, State University of New York 1084, Albany, New York, USA; 2 Molecular Epidemiology Laboratory, Federal University of Uberlândia, Uberlândia, Minas Gerais, Brazil; 3 New York State Department of Health, Wadsworth Center 1094, Albany, New York, USA; Institut National de Santé Publique du Québec, Sainte-Anne-de-Bellevue, Quebec, Canada

**Keywords:** Shiga toxin-producing *Escherichia coli *(STEC), genomics, antimicrobial resistance, serotypes

## Abstract

Shiga toxin-producing *Escherichia coli* (STEC) is a zoonotic pathogen that can be transmitted to humans through contaminated food or contact with infected animals. Here, we aim to characterize the population diversity and genome characteristics of the clinical STEC population in New York State collected over a span of six years. A total of 1,655 clinical isolates were analyzed using whole-genome sequencing, phylogenetic reconstruction, *in silico* identification of acquired antimicrobial resistance genes and plasmid types, and delineation of genetic clusters. The STEC population consists of 178 serotypes based on unique combinations of the O and H antigens. Four serotypes were most frequently detected: O103:H2 (*n* = 295 genomes), O157:H7 (*n* = 207), O26:H11 (*n* = 137), and O111:H8 (*n* = 126). The accessory genome content of O157:H7 was more divergent from those of other three serotypes. A total of 93 genetic determinants associated with resistance to 13 classes of antimicrobial agents were identified. Across the whole data set, we detected 10 subtypes of the Shiga toxin gene *stx*. The gene encoding intimin (*eae*) was present in 70.39% of the genomes. Of the 56 plasmid replicon types detected, the most abundant was IncFIB(AP001918). Using a cutoff of 10 SNPs in the core gene alignment, we identified 28 genetic clusters implicating at least ten serotypes. O157:H7 genomes were identified in eight clusters. These genetically linked cases, spanning anywhere between 1 day and 63 months and traversing multiple counties across the state, suggest either cryptic outbreaks or long-term local persistence.

**IMPORTANCE:**

Shiga toxin-producing *Escherichia coli* (STEC) infections are a major public health burden. We show that while serotypes O103:H2 and O157:H7 remain the major causes of STEC infections in New York State, the rising incidence of less common serotypes is a cause of concern, especially given the large pool of AMR, virulence genes, and plasmids they carry. Genomic surveillance of STEC is critical in informing public health efforts to reduce the burden of multidrug-resistant STEC, minimize onward transmission, and prevent more severe disease outcomes.

## INTRODUCTION

Infections caused by Shiga toxin-producing *Escherichia coli* (STEC), also known as verotoxin-producing *E. coli* (VTEC), pose a major public health burden and lead to high healthcare costs (1, 2). The intestinal tracts of ruminants, especially cattle and sheep, are the primary reservoirs of STEC where it resides as asymptomatic commensals (3). People become infected through ingestion of contaminated food or water or through direct contact with infected animals or people (4). Individuals with STEC infections exhibit typical symptoms of gastroenteritis and colitis, such as bloody diarrhea, abdominal cramps, and vomiting, that last a few days (5). It is a self-limiting disease that is managed mainly with supportive care through hydration and fluid replacement (5, 6). Historically, antimicrobials are not recommended for the treatment of STEC infection because of the possible risk of developing hemolytic uremic syndrome (7), a potentially life-threatening STEC complication that leads to serious renal failure, neurological effects, and extrarenal sequelae (8–10). However, alternative treatments such as monoclonal antibodies or antisera, vaccination, and some antimicrobial agents have shown promise against STEC (11). Other severe STEC complications include hemorrhagic colitis (inflammation of the large intestine), rare neonatal meningitis, and septicemia (5, 12, 13). Produce, beef, and dairy products are the most important food types identified as sources of globally documented STEC outbreaks (14). In the United States, the Centers for Disease Control and Prevention (CDC) collects STEC surveillance data through the Foodborne Diseases Active Surveillance Network (FoodNet). In 2022, FoodNet reported STEC infection incidence of 5.7 cases per 100,000 population, resulting in 2,882 cases of infections, 582 hospitalizations, and 11 deaths (15).

The defining characteristic of STEC is their ability to produce and release potent Shiga toxins, which inhibit protein synthesis in eukaryotic cells and induce apoptosis (16). These toxins can travel from the intestine to the bloodstream to cause damage in other major organs, such as the kidneys and brain (17, 18). The *stx* genes encoding Shiga toxins are found in the genome of the lambdoid bacteriophages, which are usually integrated as prophages into the bacterial chromosome (19). Phage-mediated horizontal transfer of *stx* genes from STEC to non-Shiga toxigenic *Enterobacter* and *E. coli* strains has been documented (20, 21).

Most STEC infections are caused by seven serogroups (O157, O26, O45, O103, O111, O121, O145), with serotype O157:H7 responsible for an overwhelming majority of outbreaks (22, 23). O157:H7 has been reported on all continents except Antarctica (24–26). Infections due to STEC O157:H7 are commonly reported in more industrialized countries (24). The ancestor of O157:H7 is inferred to have originated in the Netherlands, from which frequent inter- and intra-continental transmission events had been facilitated mainly by animal movements (24). Different classes of antimicrobials differentially influence the production of Shiga toxin in O157:H7 (27). However, STEC infections have also been attributed to more than 100 serotypes outside of the aforementioned seven, as has been observed in the United States and Europe (28–32).

Here, we aim to characterize the population structure and genome characteristics of the clinical STEC population in New York State collected over a span of six years (*n* = 1,655 genomes). We show that the STEC population is remarkably diverse in terms of genetic lineages, serotypes, and antimicrobial resistance (AMR) genes, with a few notable serotypes implicated in putative genetic clusters. Genomic surveillance of STEC is critical in informing public health efforts to reduce the burden of multidrug-resistant STEC and minimize onward transmission.

## MATERIALS AND METHODS

### Selection of bacterial isolates

New York State considers STEC infections as a reportable disease. The New York State Department of Health strongly encourages isolate or stool submission to the Wadsworth Center, the public health laboratory of the New York State Department of Health, in line with New York State’s Laboratory Reporting of Communicable Disease guidelines (https://wadsworth.org/sites/default/files/WebDoc/CDRG%20NYState%202020_101920%202.pdf). The Wadsworth Center carries out species confirmation and molecular characterization of STEC as part of New York’s bacterial foodborne outbreak detection and surveillance program. In addition, New York State is part of the CDC surveillance networks (https://www.cdc.gov/ecoli/php/surveillance/index.html), including FoodNet, which tracks trends in foodborne illnesses (15), and PulseNet, which detects disease outbreaks (33).

Stool specimens collected from individuals who had been clinically confirmed with STEC infection were received from New York State healthcare providers. However, New York State does not require healthcare providers to provide patient information, including specific disease symptoms and epidemiological information (e.g., whether the case involved food poisoning or contact with animals), to the Wadsworth Center; hence, we do not have this information to report in this study. A total of 1,763 isolates were obtained between January 1, 2018, and September 26, 2023, from 61 out of 62 counties in New York State. A total of 12 isolates did not have county information. A single isolate was obtained from each patient.

Specimen processing was described previously (29). Briefly, a portion of the submitted broth or stool was diluted 1:10 with an in-house prepared *E. coli* broth (120  g of tryptone, 30.6  g of lactose, 24  g of potassium hydrogen phosphate, 9  g of potassium dihydrogen phosphate, 30  g of sodium chloride in 6 L of Millipore H_2_O; pH 6.9) and incubated for 18–24 h aerobically at 37°C. Enriched samples were plated onto Sorbitol MacConkey agar and incubated for 18–24 h aerobically at 37°C to recover Shiga toxin-producing isolates. At least five colonies were picked per plate for STEC confirmation. All isolates were stored in glycerol solution at −80°C. Confirmation of STEC was carried out by amplification of the Shiga toxin genes (*stx1* and *stx2*), O157-specific *rfb* gene, and *wzx* or *wzy* gene for non-O157 STEC serogroups using real-time PCR assay following previously described protocol (29). The *stx* gene was detected in all isolates using real-time PCR.

Samples used in the study were subcultured bacterial isolates that had been archived in the routine course of the state public health surveillance program for foodborne illnesses. Patient protected health information was not used in this study. Therefore, informed consent was not required. This work has been determined to be exempt from human subject research by the Institutional Review Board of the Wadsworth Center.

### DNA extraction, DNA library preparation, and whole-genome sequencing

Overnight cultures were lysed using a solution of 180 µL buffer ATL and 20 µL Proteinase K (50 µg/µL) per sample for 60 min in a shaking thermomixer at 56°C. They were then placed in the QIAcube or QIAcubeHT (Qiagen, Germantown, MD), and genomic DNA was extracted using the standard QIAamp DNA Blood Minikit protocol or QIAamp 96 DNA QIAcube HT kit.

Library preparation and sequencing were carried out at the Advanced Genomic Technologies Cluster at the Wadsworth Center. DNA library preparation followed a modified Illumina DNA Prep kit using one-quarter of the recommended volume, as previously published (34). Sequencing was done using an Illumina NextSeq500. The paired-end reads (2  ×  150 bp) from the samples were classified by Kraken v.0.10.5-beta (35). The prebuilt Minikraken 20171019_8GB database was used for the reference sequence database. The Kraken output was used as input in Krona tools to generate the taxonomic assessment (https://github.com/marbl/Krona/wiki). Read quality was assessed to ensure that minimum quality thresholds established by the Center for Food Safety and Applied Nutrition (CFSAN) were met, using MicroRunQC implemented on the GalaxyTrakr platform of Galaxy (36) or thresholds established by PulseNet using Bionumerics version 7.6.3. For all genomes sequenced, *de novo* coverage was >40×, average quality (Q) score was >30, and assembly length ranged from 4.9 Mbp to 5.9 Mbp. Genome sequences were submitted to the Pathogen Detection database of the National Center for Biotechnology Information (NCBI) (https://www.ncbi.nLm.nih.gov/pathogens/) in real time.

### *De novo* genome assembly, sequence quality check, and annotation

Paired-end reads were assembled *de novo* using the Shovill v.1.1.0 pipeline (https://github.com/tseemann/shovill), which implements the k-mer–based assembly algorithm SKESA v. 2.4.0 (37) and performs pre-and post-processing by Shovill. We employed the --trim flag for trimming of adapter sequences. We used QUAST v.5.0.2 (38) and CheckM v.1.2.2 (39) to assess the quality of assembled genomes. Genomes with <90% completeness and >5% contamination were excluded from downstream analysis. We also excluded assemblies with >300 contigs and an N50 <40,000 bp to obtain high-quality genomes. After filtering low-quality genomes, we obtained a total of 1,655 genomes which were used for all downstream analyses (Table S1). Genome completeness ranged from 98.28 to 99.66% (median = 99.64%), and genome contamination ranged from 0.099 to 1.82% (mean = 0.428%), which were all within the genome quality standards recommended by CheckM (39). The number of contigs in our data set ranged from 28 to 300 (median = 176), and N50 ranged from 40,107 to 560,636 bp (mean = 131,115.8 bp). Genome sizes ranged from 4.712 to 5.889 Mbp. To confirm species identity, all the genomes were compared to the reference genome *E. coli* strain K-12 substrain MG1655 (NCBI Accession no. GCA_000005845.2) using fastANI (40), applying the standard >95% average nucleotide identity (ANI) threshold for species delimitation (Table S1). The resulting contigs were annotated using Prokka v.1.14.6 (41).

### Pan-genome analysis and phylogenetic reconstruction

We used Panaroo v.1.3.4 (42) to characterize the collective set of genes present in all genomes in our data set, i.e., pan-genome (43). We used the flag --strict option to ensure that only high-quality gene sequences were identified and clustered. The pan-genome consisted of 23,708 orthologous gene families. These genes were categorized as core genes (*n* = 3,182; genes present in 99% of genomes), softcore genes (*n* = 338; genes present in 95 to <99% of genomes), shell genes (*n* = 2,964; genes present in 15 to <95% of genomes), and cloud genes (*n* = 17,224; genes present in <15% of genomes) (Table S2). The number of genes per genome ranged from 4,373 (Accession number SRR13165659; isolate from Chautauqua County in November 2020) to 5,952 (Accession number SRR25463564; isolate from St. Lawrence County in July 2023). The mean number of genes per genome was 5,210.61 ± 264.27 (standard deviation). Nucleotide sequences were aligned using MAFFT v.7.471 (44).

Single nucleotide polymorphisms (SNP) were extracted from the 3.615 Mbp concatenated alignment of 3,182 core genes using SNP-sites v.2.5.1 (45). The core gene alignment consisted of 533,511 SNPs, which was used as input in IQ-TREE v.2.1.4 (46) to build a maximum likelihood phylogeny. We used the ModelFinder algorithm to determine the best-fit model for ascertaining rate heterogeneity, improving the accuracy of phylogenetic estimates (47). We used the general time-reversible nucleotide substitution model (48) with an ascertainment bias correction and discrete Gamma model with default of four rate categories (GTR + ASC + G). Branch support was assessed using 1,000 bootstrap replicates, implemented using the built-in ultrafast bootstrap (UFBoot) method (49). Phylogenetic trees were rooted at the midpoint. Trees were visualized and annotated using the Interactive Tree of Life (50).

To assess dissimilarities in the accessory genome composition among the most frequent *E. coli* serotypes, a Jaccard distance analysis was performed using the gene presence/absence matrix generated by Panaroo (42). The Jaccard distance was computed using the distJaccard function from the micropan v.2.1 (51) package in R v.4.4.2 (52). We also carried out a principal coordinates analysis (PCoA) using the cmdscale function from the stats package (https://stat.ethz.ch/R-manual/R-devel/library/stats/html/00Index.html), implementing three dimensions (k = 3) to visualize genetic dissimilarity among genomes. The variance explained by each axis was calculated based on the resulting eigenvalues. To evaluate the statistical significance of observed differences between serotypes, a PERMANOVA test with 999 permutations was performed using the adonis2 function from the vegan v.2.6-10 package (53). Pairwise post-hoc comparisons between all serotype combinations were conducted using the same permutation approach to identify specific group differences.

### *In silico* identification of serotype, phylogroup, sequence type (ST), antimicrobial resistance (AMR) genes, and mobile genetic elements

The ST of each isolate was determined using mlst v.2.19.0 (https://github.com/tseemann/mlst), which extracts the sequences of seven housekeeping genes (*adk*, *fumC*, *gyrB*, *icd*, *mdh*, *purA*, *recA*) based on the Achtman MLST scheme (54) from the Illumina raw data and compares them to allelic profiles available in the *E. coli* ST database on pubMLST (55). We identified 19 genomes lacking assigned STs that were submitted to EnteroBase (56) for validation, resulting in the assignment of 16 new STs (ST17901 to ST17917; Table S1). Phylogroups were identified using ClermonTyping with default parameters (57).

To identify AMR determinants, we ran AMRfinderPlus (58) using thresholds of 60% sequence coverage and 80% sequence identity. To determine the serotypes defined by the O (lipopolysaccharide, LPS) and H (flagellar) antigens, we used ABRicate v.0.8.10 (https://github.com/tseemann/abricate) and the EcOH database (59) and maintained a conservative approach implementing a 100% sequence identity threshold for the O and H identification. Genomes lacking one or both assigned antigen(s) were submitted to the EnteroBASE module *EBEis* for further characterization (60). We determined the presence of virulence genes using Abricate with the ecoli_vf database (https://github.com/phac-nml/ecoli_vf) implementing query thresholds of 60% sequence coverage and 80% sequence identity. Within the ecoli_vf database, we also identified key genes associated with siderophore systems. Specifically, we determined the presence of the complete operons for aerobactin (*iucABCD*), enterobactin (*entEBGAC*), salmochelin (*iroBCDEN*), and yersiniabactin (*ybtAEPQSTUX*, *irp1*, *irp2*, *fyuA*). To identify the *stx* subtypes of each genome, we used StxTyper v.1.0.27 (https://github.com/ncbi/stxtyper), implementing thresholds of 60% sequence coverage and 80% sequence identity for each gene. StxTyper uses a reference database of StxA and StxB protein sequences from the NCBI Pathogen Detection Reference Gene Catalog (https://www.ncbi.nlm.nih.gov/pathogens/refgene), and only query sequences that include both genes are classified at the subtype level. Genome sequencing confirmed the presence of the *stx* genes detected using PCR, except for two genomes (SRR24478418 and SRR7958545), likely due to the *stx* gene missing from fragmented short-read sequences or that the gene is split across different contigs. We used Abricate with the PlasmidFinder database and default parameters to carry out an *in silico* detection and identification of putative plasmids based on the presence of the replicon gene (*rep*), which encodes the plasmid replicon initiator protein (61).

### Identification of genetically linked clusters

For each pair of genomes, we calculated the genetic distance based on SNPs in the concatenated alignment of core genes using snp-dists v.0.8.2 (https://github.com/tseemann/snp-dists). A threshold of ≤10 SNP differences between genomes was applied to define genetic clusters, a criterion previously established as indicative of epidemiological linkage in *E. coli* (62–65). For the purposes of this study, a cluster was defined as a group comprising three or more genomes. Such clusters likely represent isolates originating from a common source of contamination or reflecting direct transmission chains. The core gene alignment generated by Panaroo was used as input for GrapeTree v.2.1 to construct minimum spanning trees of the core SNP clusters (66).

### Statistical analysis

All statistical analyses were carried out using ggstatsplot v.0.13.0 (67) and plots created using ggplot2 v.3.5.1 (68), both of which are implemented in R v.4.4.2 (52). Kruskal-Wallis test with Holm-Bonferroni correction was used for comparison of the number of plasmids, AMR determinants, and virulence genes per genome between serotypes. A *P*-value threshold of ≤0.05 was used to determine statistical significance.

## RESULTS

### Population structure of STEC in New York State

**Fig 1:**
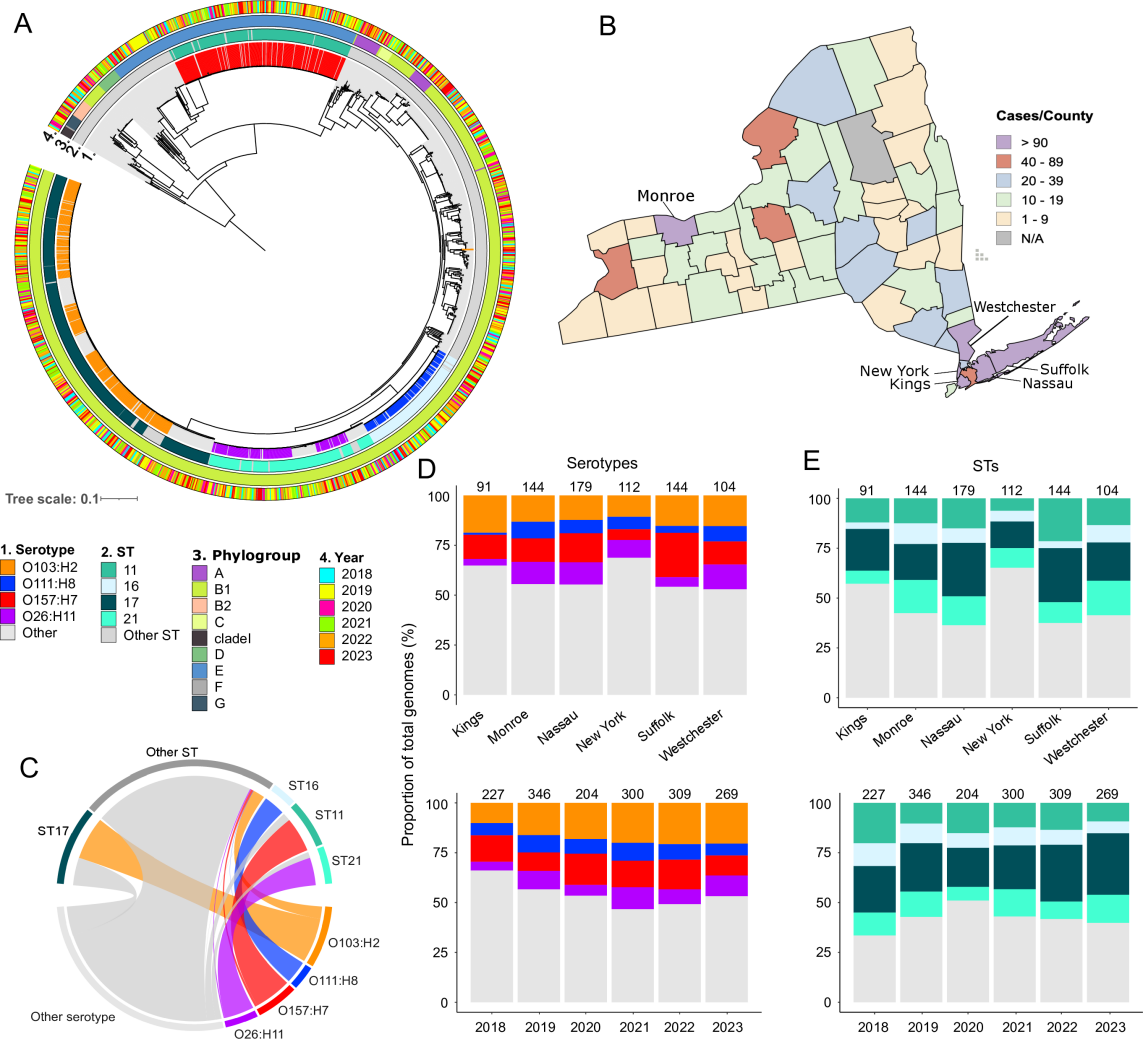
Population genomic structure of clinical STEC (*n* = 1,655 genomes) isolates in New York State. (**A**) Midpoint-rooted maximum likelihood phylogenetic tree built from the sequence alignment of 533,511 single-nucleotide polymorphisms in 3,182 core genes. Tree scale represents the number of nucleotide substitutions per site. Outer rings show the year of sampling, serotype, sequence type (ST), and phylogroup. For visual clarity, only the most common categories of serotypes and STs are color-coded, and less common types are grouped into “Others.” (**B**) Geographical distribution of genomes. Six of the 62 counties with the highest number of STEC genomes are labeled and indicated by arrows. (**C**) Chord diagram showing the distribution of the main serogroups by ST. Distribution of the (**D**) four most common serotypes and (**E**) four most common STs according to county (top plot) and year (bottom plot). In panel D, numbers above the bars indicate the total number of genomes in each county over the entire sampling period. In panel E, numbers above the bars indicate the total number of genomes across per year all counties. Details of the genomic features of individual isolates are presented in Table S1.

We analyzed 1,655 short-read genomes derived from STEC isolates that were received from New York healthcare providers (Table S1). The core genome maximum likelihood tree built from 533,511 SNPs extracted from the concatenated alignment of 3,182 core genes reveals their phylogenetic relationships. There are at least four large phylogenetic clusters, with each one derived from short branches and consisting of very closely related genomes (Fig. 1A). Genomes from different years are intermingled with each other. The STEC genomes were obtained from 61 of the 62 counties of New York State, with six counties (Kings, Monroe, Nassau, New York, Suffolk, and Westchester) having the highest number of genomes (Fig. 1B). However, we note that the number of genomes per year and per county varied greatly, and inferences about the STEC distribution in New York State should be made only within the context of this current data set.

The STEC population in New York State is remarkably diverse. We identified 178 serotypes based on *in silico* detection of unique combinations of the O and H antigens. Four serotypes were frequently detected: O103:H2 (*n* = 295 genomes, 17.82% of the data set), O157:H7 (*n* = 207, 12.51%), O26:H11 (*n* = 137, 8.28%), and O111:H8 (*n* = 126, 7.61%) (Fig. 1A and C). The four dominant serotypes were consistently present in the six aforementioned counties and in the six years of sampling (Fig. 1D). Serotype O103:H2 was the most widely distributed and was identified in 52 counties.

Multilocus sequence typing (MLST) based on allelic variation in seven single-copy housekeeping genes (54) revealed 175 previously recognized STs. We identified 16 new STs (ST17901 to ST17917; Table S1). Four STs were most frequently detected: ST17 (*n* = 414 genomes, 25.02% of data set), ST11 (*n* = 217, 13.11%), ST21 (*n* = 190, 11.48%), and ST16 (*n* = 141, 8.52%). The four dominant STs were consistently present in the six counties and in the six years of sampling (Fig. 1E). Not one ST was identified in all counties, but ST17 and ST11 were present in 53 and 51 counties, respectively. A total of 84 STs were represented by a single isolate.

The four major serotypes are strongly linked to the four predominant STs (Fig. 1C). In particular, 259 genomes (87.80%) of serotype O103:H2 belonged to ST17, with 292 genomes (98.98%) assigned to Clonal Complex (CC) 17. Similarly, most genomes of serotype O157:H7 were classified as ST11 (195 genomes; 94.20%) and CC11 (203 genomes; 98.97%). The O111:H8 serotype was predominantly associated with ST16 within CC29 (124 genomes; 98.41%), whereas the O26:H11 serogroup comprised 127 genomes (92.70%) related to ST21, with 132 genomes (96.35%) also belonging to CC29.

We identified nine phylogroups, of which B1, E, and A were the most frequently detected, representing 94.86% of the data set (*n* = 1219, 296, and 55 genomes, respectively; Fig. 1A). Phylogroup B1 was the most widely distributed and was identified in 59 counties. B1 also contained the most common STs and serotypes, including O111:H8, O26:H11, O118:H2, and O103:H2. Serotype O157:H7 genomes were assigned to phylogroup E (Table S1). Some of the phylogroups were intermingled (A and C within B1), as has been observed in previous studies (69–71). We also detected the presence of 10 genomes representing the recently identified cryptic C-I (clade I), which is recognized as an emerging source of human intestinal pathogens that likely originated from bovine sources (72).

Since 2019, *E. coli* O103:H2 has become the most frequent serotype in New York, surpassing O157:H7 (Fig. S1A). To examine the genomic characteristics of this serotype relative to the others, we analyzed the accessory genome variation by calculating Jaccard distances among the four most frequent serotypes and the remaining genomes (classified as “Other” in Fig. 1). The PCoA revealed partial separation among serotypes (PCo1: 17.9%; PCo2: 13.9%) (Fig. S1B). O157:H7 exhibited the most distinct accessory genome profile, whereas O103:H2 was closer to O111:H8 and O26:H11 (pairwise *R*² = 0.59 for both), suggesting that O103:H2 may share accessory genes with other common serotypes while remaining distinct from O157:H7. A PERMANOVA (Table S3) confirmed that serotype explained a significant proportion of accessory genome variation (*R*² = 0.34, F = 215.9, *P* = 0.001), with all pairwise comparisons significant (*P* = 0.001). The strongest differences involved O157:H7 (pairwise *R*² = 0.71–0.80), indicating a highly distinct accessory genome.

### O103:H2 harbors greater diversity of AMR determinants, whereas O157:H7 has higher number of virulence genes per genome

**Fig 2:**
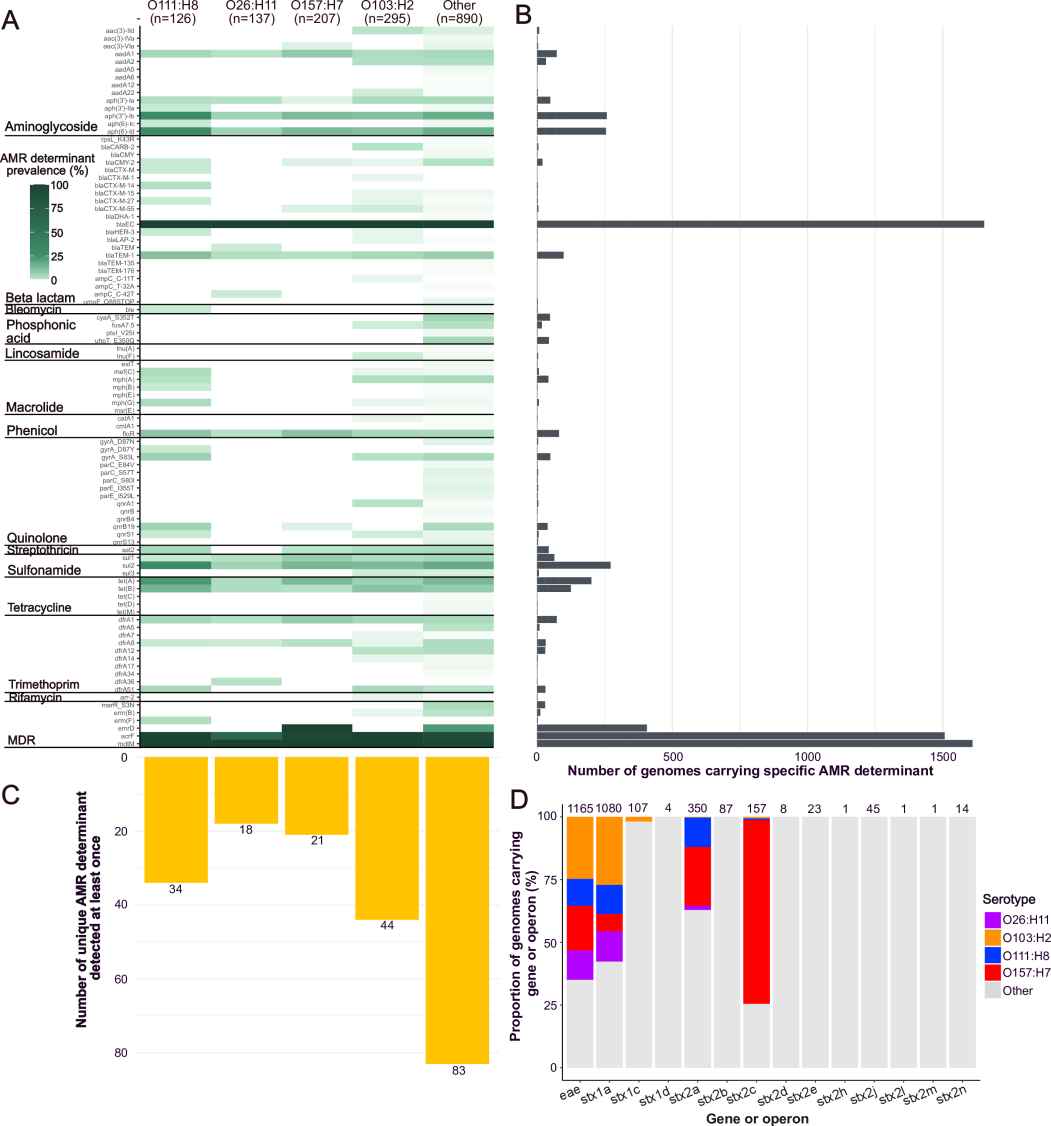
Distribution of acquired antimicrobial resistance (AMR) and virulence genes. (**A**) Prevalence of an AMR gene (row) was detected in a specific serotype (column) out of the total number of genomes of the serotype. For visual clarity, only the most common AMR genes and only the four most dominant serotypes are shown. Less common serotypes are grouped together in the category “Others.” (**B**) Total number of genomes carrying a specific AMR gene. (**C**) Total number of AMR genes detected at least once per serotype. (**D**) Proportion of genomes carrying the virulence genes *eae*, *stx*, and *stx* subtypes. For visual clarity, only the four most dominant serotypes are shown. Less common serotypes are grouped together in the category “Others.” Numbers above the bars indicate the total number of genomes that carry the gene or gene variant. Colors of the serotypes correspond to those in Fig. 1. Details of the distribution of all AMR and virulence genes among individual isolates are presented in Tables S4 and S5, respectively.

Using *in silico* screening of all 1,655 STEC genomes, we identified 93 unique determinants conferring resistance to 13 classes of antimicrobial agents as well as genes conferring multidrug resistance (Fig. 2A; Table S4). The number of AMR genes per genome ranged from 2 to 18. No AMR determinant was universally present across all analyzed genomes (Fig. 2B). The *blaEC* gene, associated with cephalosporin resistance, was the most frequent and was detected in 1,653 genomes (99.88%). This was followed by *mdtM* and *acrF*, genes related to efflux pumps and multidrug resistance, which were present in 1,610 (97.28%) and 1,507 (91.06%) genomes, respectively. All other AMR determinants occurred in fewer than 50% of the genomes.

There are some notable AMR determinants that are worth highlighting. The *ermD* gene, associated with multidrug resistance including resistance to phenolic antibiotics and disinfectant agents, was identified in 406 genomes (24.53%). Genes associated with sulfonamide resistance, *sul2* and *sul1*, were detected in all major serotypes, present in 272 (16.44%) and 64 (3.87%) genomes, with *sul2* being the fifth most frequent. Aminoglycoside-related genes *aph(3″)-Ib* and *aph (6)-Id*, also identified in all major serotypes, ranked as the sixth (258 genomes; 15.59%) and seventh (255 genomes; 15.41%) most frequent, respectively, followed by *tet(A*) and *tet(B*), associated with tetracycline resistance and present in 201 (12.15%) and 125 (7.55%) genomes. Other genes identified across all serotypes included aminoglycoside-associated genes *aadA1* (73–4.41%) and *aph(3′)-Ia* (49 genomes; 2.96%), beta-lactamase gene *blaTEM-1* (98 genomes; 5.92%), phenicol resistance gene *floR* (81 genomes; 4.89%), and trimethoprim resistance genes *dfrA1* (73 genomes; 4.41%) and *dfrA8* (31 genomes; 1.93%).

Genomes that belong to less common serotypes (represented as “Other” in Fig. 1A) exhibited the highest number of unique AMR determinants per genome (83 – 89.25%) (Fig. 2C). Among the major serotypes, O103:H2 displayed the second highest diversity of AMR determinants (44–47.31%), including genes conferring resistance against aminoglycoside (*aac (3)-IId*, *aadA2*, *aadA22*), beta-lactams (*blaCARB-2*, *blaCMY-2*, *blaCTX-M-1/15/27/55*, *blaHER-3*, *blaLAP-2*, *ampC* C11T), fosfomycin (*fosA7.5*), lincosamide (*lnu(F*)), phenicol (*catA1*), quinolones (*gyrA* S83L, *qnrA1/S1*), sulfonamides (*sul3*), trimethoprim (*dfrA7/8/12/14/51*), rifampicin (*arr-2*), and multidrug resistance (*erm(B*)) determinants. Serotype O111:H8 harbored 34 (36.17%) AMR determinants representing all antimicrobial classes, including genes conferring resistance to aminoglycoside (*aph(3′)-IIa*, *aph (6)-Ic*), beta-lactamases (*blaCMY-2*, *blaCTX-M-14/27*, *blaHER-3*), bleomycin (*ble*), macrolides (*mef(C)*, *mph(ABG)*), quinolones (*gyrA D87Y/S83L*, *qnrB19/S1*), trimethoprim (*dfrA51*), and multidrug resistance (*erm(F*)) determinants. The presence of extended-spectrum beta-lactamases (ESBLs) in these serotypes is alarming, as these broad-spectrum enzymes confer resistance to penicillins and cephalosporins and efficiently hydrolyze oxyimino-beta-lactams, including cefotaxime, ceftazidime, and aztreonam (73). Serotype O157:H7 contained 21 (22.34%) AMR genes and lacked resistance determinants against fosfomycin, lincosamides, macrolides, or bleomycin. O26:H11 possessed 18 (19.15%) genes and lacked the fosfomycin, lincosamide, bleomycin, or quinolone resistance mutations. Despite these differences, the median number of AMR determinants per genome ranged between three and four, with O157:H7 exhibiting a higher median (median = 4, Kruskal-Wallis *P* < 0.05; Fig. S2A).

We also identified the presence of virulence genes pertinent to STEC infection. Serotype O157:H7 harbored the highest number of virulence genes per genome (median = 300) compared to other serotypes, followed by O103:H2 (median = 273) (Kruskall-Wallis, *P* < 0.05, Fig. S2B). PCR confirmed the presence of the *stx* gene in all isolates. To determine the *stx* subtype, a genome was considered positive for a given type only when the complete operon (subunit A and subunit B) was present. Using this criterion, we identified *stx* subtypes in 1,635 genomes (Table S5). In 19 genomes, only partial genes related to either subunit A or subunit B were detected. Each genome carried up to two *stx* subtypes. We identified three *stx1* subtypes, with *stx1a* being the most frequent (1,080 genomes; 62.26%), followed by *stx1c* (107 genomes; 6.46%) and *stx1d* (four genomes; 0.24%). Among the major serotypes, all contained genomes with *stx1a*, but only O103:H2 carried genomes with *stx1c*, and none harbored *stx1d*. For *stx2*, the most frequent subtype was *stx2a* (350 genomes; 21.15%), followed by *stx2c* (157 genomes; 9.49%). These were the only *stx2* subtypes detected across all four major serotypes, except for serotype O26:H11, which lacked *stx2c*. Among the remaining *stx2* subtypes, the most common were *stx2b* (87 genomes; 5.26%), *stx2j* (45 genomes; 2.72%), *stx2e* (23 genomes; 1.39%), *stx2n* (14 genomes; 0.85%), *stx2d* (eight genomes; 0.48%), and *stx2h*, *stx2l,* and *stx2m* (one genome each; 0.06%). Across the entire data set, we also found the *eae* gene in 70.39% of the genomes (Table S5). The *eae* gene encodes the outer membrane adhesion protein called intimin that functions in attaching and effacing lesions and thus plays a role in intestinal colonization (74). The *eae* gene was detected in all or nearly all genomes of the four major serotypes (O26:H11 [137 genomes; 100%], O103:H2 [288 genomes; 97.74%], O111:H8 [125 genomes; 91.67%], and O157:H7 [207 genomes; 100%]) as well as in less common serotypes.

We also investigated siderophore-associated genes and identified the presence of enterobactin in all major and less common serotypes. Aerobactin was detected in all serotypes except O157:H7. Yersiniabactin was found in the majority of O26:H11 genomes (133–97.08%) and at lower frequencies in other less frequent serotypes (145–16.29%). Salmochelin was identified in a small subset of the less frequent serotypes (40–4.49%). The ability to scavenge the essential micronutrient iron through siderophores is critical to *E. coli* growth and survival, thus facilitating gut colonization (75).

### STEC carries a large pool of plasmid replicon types

**Fig 3:**
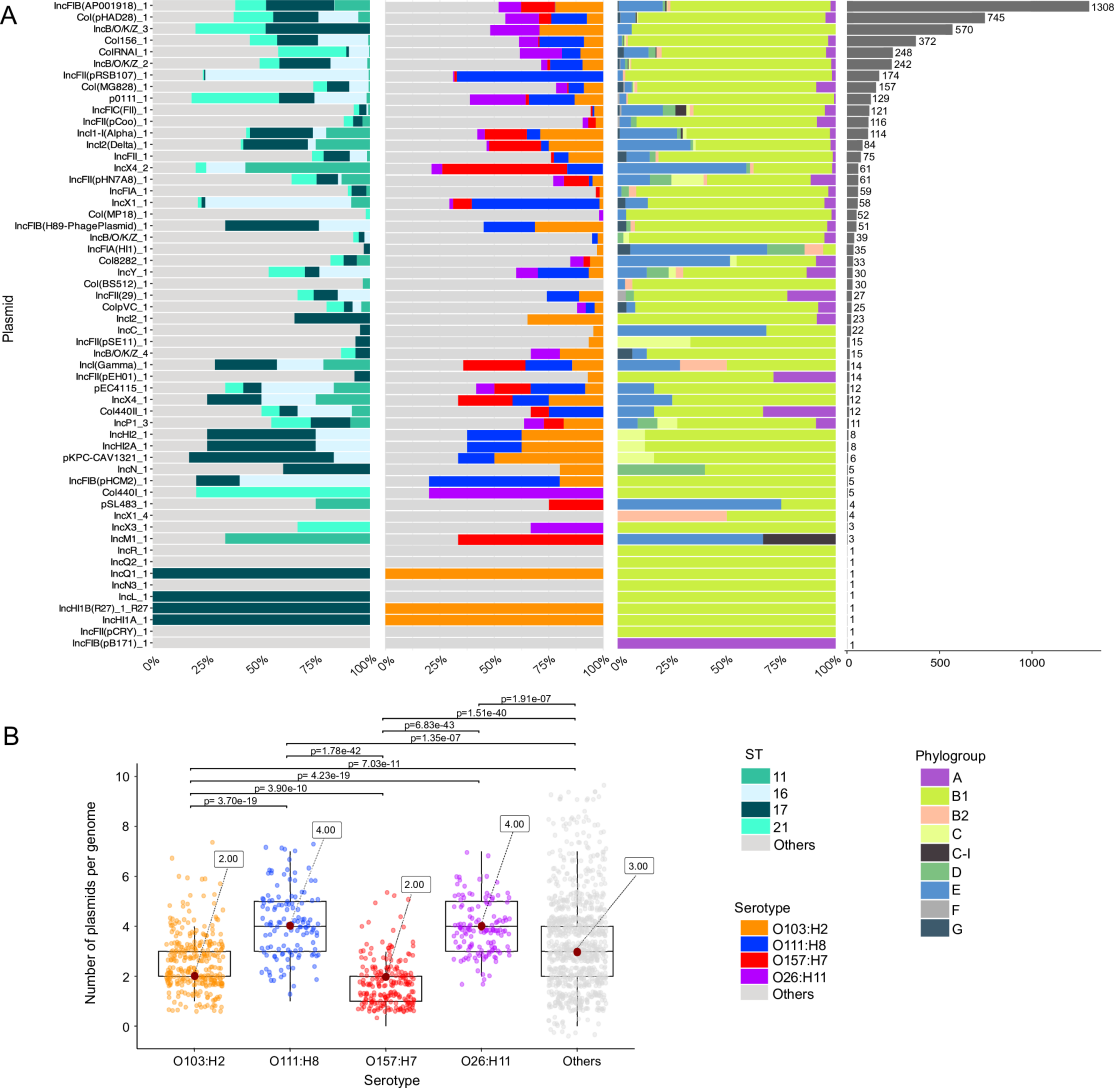
Distribution of putative plasmid replicon types. (**A**) The abundance of each plasmid type is shown as a proportion of their presence in different STs (left bar plot), serotypes (middle bar plot), and phylogroups (right bar plot). The fourth bar plot shows the total number of sequences of each plasmid replicon type that were detected. For visual clarity, only the most common STs and serotypes are shown. (**B**) Comparison of the number of putative plasmid replicon types per genome among the four most common serotypes and other serotypes (Kruskal-Wallis test with Holm-Bonferroni correction). Less common STs and serotypes are grouped together in the category “Others.” Box and whisker plots show the distribution of data into quartiles, median, variability outside the upper and lower quartiles, and outliers. Jitter dots represent individual genomes. The numbers inside the boxes show the median values. Details of the distribution of plasmids replicon types among individual isolates are presented in Table S6.

We sought to identify the presence of putative plasmids by typing variants of the replicon gene (*rep*), which encodes the plasmid replicon initiator protein. We identified a total of 56 replicon types present across the entire STEC population, of which the most abundant was IncFIB(AP001918) that was present in 1,308 (79.03%) genomes (Fig. 3A; Table S6). This plasmid was detected in all four major STs and four major serotypes (O103:H2, O111:H8, O157:H7, O26:H11). Three other plasmid types were also frequently found and included CoI(pHAD28), INCB/O/K/Z, and CoI156 that were detected in 745 (45.01%), 570 (34.44%), and 372 (22.48%) genomes, respectively.

A total of 1,636 genomes (98.85% of the data set) carried at least one plasmid replicon type. Among the major STs, we detected the presence of 27, 27, 42, and 30 distinct plasmid types in ST11, ST16, ST17, and ST21, respectively. Among the major serotypes, we identified 40, 27, 23, and 24 distinct plasmid types in O103:H2, O111:H8, O157:H7, and O26:H11, respectively. Phylogroup B1 contained the largest variety of plasmid types (54 of 56 types). A total of 19 plasmid types were rarely detected and were found in fewer than 10 sequences. Numerous rare STs and serotypes harbored a wide variety of plasmid replicon types. We found significant differences in the number of plasmid replicon types per genome among the serotypes (Fig. 3B; all paired grouped comparisons with *P* < 0.05, Kruskal-Wallis test with Holm-Bonferroni correction).

### Diverse serotypes are associated with genetically linked clusters

**Fig 4:**
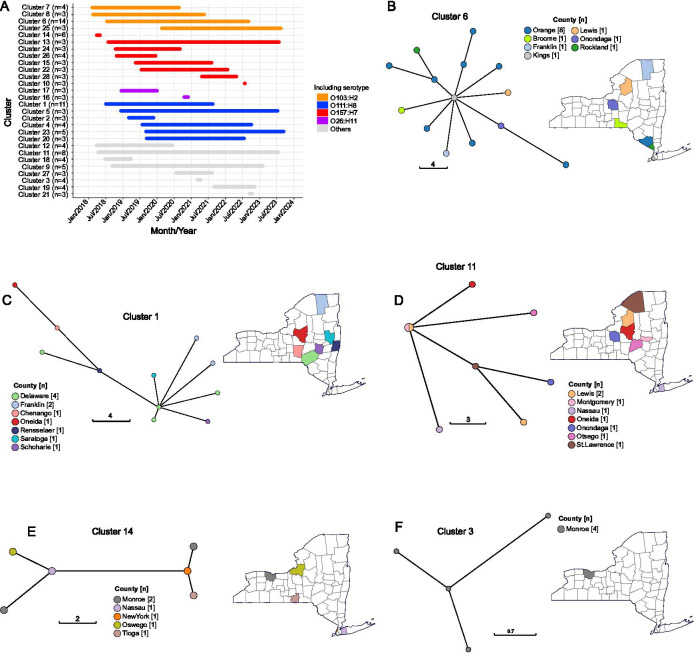
Genetic clusters based on pairwise differences of ≤10 SNPs in the core gene alignment. Only those sequence clusters with at least three genomes are shown. (**A**) The 28 clusters show the number of genomes which are indicated by *n*. Time of sampling (month and year) is shown on the x-axis. The length of the horizontal lines represents the duration of time which the isolates were collected. Horizontal lines of the same color are part of the same serotype. Highlighted here are the larger sequence clusters of the common serotypes (**B**) O103:H2, (**C**) O111:H8, (**D**) O121:H19, (**E**) O157:H7, and (**F**) O145:H28. Evolutionary relationships are shown as minimum spanning trees. The scale represents the number of SNPs, and the length of the scale is proportional to the number of SNP differences. The size of nodes is proportional to the number of genomes. The number in brackets next to the county name indicates the number of genomes. For panels **B–F**, nodes of the minimum spanning trees and counties on the maps are colored by county of isolation. The remaining clusters, including their acquired antimicrobial resistance genes, are presented in Table S7.

Using a threshold of ≤10 SNPs in the core gene alignment to define genetic clusters (62–65), we identified 28 clusters implicating 10 serotypes (Fig. 4A; Table S7). Here, we only included those clusters consisting of at least three genomes. Of the 28 clusters, 20 involved the four major serotypes: O103:H2, O111:H8, O157:H7, and O26:H11. Eighteen clusters spanned multiple years, ranging from 13 to 63 months, suggesting long-term local persistence in the population. The clusters harbored between 3 and 16 AMR determinants and contained 1 to 11 plasmid replicon types, which were identified in at least one genome. All genetic clusters contained the *blaEC* and *mdtM* resistance genes, which were detected in at least one genome of each cluster.

Across the entire data set, we identified 59 genomes that were linked to foodborne disease outbreaks reported in the CDC PulseNet database (https://www.cdc.gov/pulsenet/hcp/about/index.html) (33), of which 22 genomes were part of seven of the 28 genetic clusters (Table S7; Fig. S3). Among these genetic clusters, three comprised O157:H7 genomes, while one cluster each consisted of O103:H2, O145:H28, O26:H11, and O45:H2 genomes. Here, we highlight the five largest genetically linked clusters (Fig. 4B through F).

The largest genetic cluster was cluster 6 (Fig. 4B) and consisted of 14 genomes of serotype O103:H2 and ST17 (Fig. 4B; Table S7). Genomes in this cluster were derived between July 2018 and March 2023 across seven counties. Four genomes from this cluster were linked to a CDC PulseNet-defined multi-state outbreak comprising eight isolates. Cluster 6 harbors seven unique AMR determinants and 10 plasmid replicon types detected at least once. We identified the plasmid replicon types IncFIB(AP001918)_1 and IncB/O/K/Z_3 in 14 genomes. A single genome harbored genes conferring resistance to aminoglycosides (*aph(3′′)-Ib, aph (6)-Id*) and sulfonamides (*sul2*), while two genomes carried the beta-lactam resistance gene *blaTEM-1*.

Two of the largest genetic clusters were not linked to any outbreaks reported in the CDC PulseNet database. Cluster 1 (Fig. 4C) comprised 11 genomes of serotype O111:H8 (ST16), which originated from seven counties over a three-year period (July 2018 to August 2021). This cluster harbored seven plasmid replicon types and three AMR determinants. Cluster 11 (Fig. 4D), consisting of eight O121:H19 (ST655) genomes, was isolated from a similarly wide geographic range of seven counties over a five-year span (April 2018 to July 2023). We identified three plasmid replicons and three AMR determinants in this cluster.

Cluster 14 (Fig. 4E), comprising six genomes, included serotype O157:H7 and sequence type ST11. These genomes originated from five counties between March and May 2018. Cluster 14 genomes were associated with a CDC PulseNet-defined outbreak that consisted of 12 isolates. We identified two plasmid replicon types and 12 antimicrobial resistance determinants. The plasmid replicon type IncFIB(AP001918)_1 was detected in all genomes. All six genomes also carried genes conferring resistance to aminoglycosides (*aadA1*, *aph(3′′)-Ib*, *aph (6)-Id*), diaminopyrimidines (*dfrA1*), phenicols (*floR*), sulfonamides (*sul1, sul2*), and tetracyclines (*tetA*), as well as the multidrug efflux pump *emrD*.

Finally, Cluster 3 (Fig. 4F) comprised four O145:H8 genomes belonging to ST32. This cluster was geographically restricted to a single city (Monroe) and exhibited a narrow isolation date range of one day. According to the CDC PulseNet outbreak database, this cluster was part of a larger multistate outbreak, which included 16 isolates. We identified four plasmids in all four genomes (Col8282_1, IncB/O/K/Z_3, IncC_1, IncFIB(AP001918)_1) and 14 AMR determinants. Three of the four genomes contained genes conferring resistance to aminoglycosides (*aadA2*, *aph(3′)-Ia*, *aph(3′′)-Ib*, *aph (6)-Id*), beta-lactams (*blaCMY-2*), macrolides (*mph(A*)), phenicols (*floR*), sulfonamides (*sul1*, *sul2*), tetracyclines (*tet(A*)), and trimethoprim (*dfrA12*), in addition to the multidrug resistance efflux pump gene *emrD*.

## DISCUSSION

STEC remains a formidable threat to public health. In this genomic study of clinical STEC that were sampled as part of a statewide surveillance program of foodborne bacterial diseases, we highlight two main findings. First, the STEC population in New York State is remarkably diverse in terms of genetic lineages, serotypes, AMR genes, and plasmid types. Serotype O157:H7 continues to be an important cause of STEC infections in the past six years, but numerous non-O157 serogroups are certainly a growing concern. Second, genetic clusters delineated using core gene SNPs traverse multiple counties and ranged from 1 day to 63 months. Genetically linked cases may suggest either cryptic outbreaks (occurring for a few months) or multi-year persistence of specific STs, serotypes, and AMR determinants across the state.

STEC surveillance in New York State using culture-independent diagnostic tests and real-time PCR implemented since 2011 already revealed the growing importance of non-O157 STEC (29). These findings are consistent with previous studies showing increasing trends in the prevalence and incidence of non-O157 STEC and outbreaks as reported in other parts of the United States (30, 76, 77) and globally (23, 32, 78). The growing number of non-O157 STEC infections is attributed in part to international travel and domestic exposure to various foods and farm animal environments (79). Our genomic analyses further contribute to the current knowledge about the diversity and distribution of non-O157 STEC. Uncommon serotypes are likely important couriers of rare AMR determinants, mobile genetic elements, and novel subtypes of *stx* genes that circulate across the entire population.

The high frequency of O103:H2 identified in our study, surpassing even O157:H7, is intriguing. This finding contrasts with national data from the CDC’s National Outbreak Reporting System (NORS), which recorded O157:H7 between 2018 and 2023 and which has the highest number of isolates linked to multi-state outbreaks in the United States, followed by O103:H2, O26:H11, O121:H19, and O111:H8 (https://www.cdc.gov/ncezid/dfwed/BEAM-dashboard.html). During this period, 21 new outbreaks were detected in New York State. Our observation aligns with the shifting serotype profile documented in New York State. While O157 predominated between 2005 and 2010 (28), O103 became more frequent from 2011 to 2022 (29). In other countries, such as England and Wales, a decline in O157:H7 and an annual increase in O103:H2 were also reported between 2014 and 2022 (80). Furthermore, studies from other countries reinforce the heterogeneity in serogroup prevalence. In Canada (2018–2021), O26 was most frequently detected, followed by O103 and O111 (81). In Germany, serotype O26:H11 led among clinical isolates between 2020 and 2022 (82).

*E. coli* has an open and dynamic pangenome with its accessory gene diversity being essential for adaptation to different environments and hosts (83). Our results demonstrate that serotype O157:H7 presents a highly divergent accessory genome compared to the other serotypes, possessing an abundance of virulence genes and higher frequency of *stx2* subtypes, which may explain its historical predominance and greater clinical severity and complications such as hemolytic uremic syndrome (7). Such a divergent virulent profile was also documented in STEC populations colonizing bovine hosts (84). However, its unique gene combination may likely restrict it to more limited ecological niches. In contrast, the genomic plasticity of O103:H2 and O26:H11 may explain their high frequency in humans and their distribution across diverse niches. This characteristic confers remarkable ecological and adaptive versatility. O103 and O26 are not only the most prevalent serogroups in cattle in North America (85), but have also been detected in a wide variety of animal hosts and food products worldwide (reviewed in references 23, 86). Moreover, O103:H2, O26:H11, and O111:H8 genomes in our study carry siderophores, such as aerobactin and yersiniabactin, which are absent in O157:H7 isolates and may favor iron acquisition and survival in multiple hosts (75). Recent studies have demonstrated that serotype O103:H2 is capable of surviving in dried mixed biofilms for extended periods (up to 60 days at 25°C) (87). This persistence capacity includes maintained viability even in fermented foods for long periods of time (88). These features are likely associated with their large accessory genomes and are also relevant to transmission. This broad host diversity—combined with their presence in cattle, the main transmission source to humans (89)—likely increases the number of infection sources and contributes to the emergence of these serotypes as pathogens of growing public health concern.

Another factor contributing to the high frequency of O103:H2 is the repertoire of AMR determinants, which may favor its persistence in the population over many months. This diversity includes the presence of genes encoding various ESBLs, which confer resistance to a broad range of beta-lactam antibiotics, including cephalosporins and oxyimino-beta-lactams, and therefore poses significant challenges for clinical management. This has been observed for multidrug-resistant *E. coli*, which can persist in livestock production environments, underscoring the potential for environmental reservoirs to contribute to the maintenance and spread of resistance to humans (90, 91). In addition, the high variety of plasmids found in this serotype likely facilitates the dissemination of these genes. Genetic determinants of AMR can be mobilized not only between *E. coli* strains (92, 93), but also between *E. coli* and other bacterial species (94), further aggravating the widespread dissemination, risks, and burden of multidrug resistance.

Our genetic cluster analysis involved all of the four most frequent serogroups, as well as O45:H2, O118/O151:H2, O121:H9, O123/O186:H2, and O145:H28. Of these, six serotypes (O145:H28, O103:H2, O157:H7, O26:H11, and O45:H2) were related to known multistate outbreaks in the United States between 2018 and 2023. The genetic features that contribute to the success of non-O157 serotypes vary. For O103:H2 STEC, it may be due partly to the recombination-driven genetic mosaicism of Shiga toxin-encoding phages in bovine and human strains (95). For O26:H11, it may be attributed to the recent and repeated acquisition of the *stx2* gene and the accumulation of more AMR genes (96). For O121:H19, different lineages exhibit unique evolutionary histories characterized by the independent acquisition of virulence plasmids, variation in the prophage pool, and prophage-encoded type III secretion systems (97). The virulence gene *eae* encoding intimin in O111:H8, carried by the locus of enterocyte effacement pathogenicity island, is genetically mosaic consisting of divergent segments that differ from those found in other *E. coli* strains (98). Regardless of the differences in the underlying causes, the increasing proliferation of non-O157 serotypes is not negligible and should be closely monitored over the long term.

A robust surveillance program of STEC is key to early detection of emerging high-risk strains, source traceback, and outbreak containment. Whole-genome sequencing of a large number of isolates from various time periods and locations is critical to uncover likely origins of clustered isolates and routes of geographical spread. Beyond surveying serotypes and STs, it is also important to acknowledge that certain genetic elements may exhibit routes of dissemination different from the cells that harbor them. Accessory genome epidemiology (99) and plasmid epidemiology (100) are nascent branches of the broader field of infectious disease epidemiology, which recognize the rapid gene turnover via gene gain and loss in bacterial genomes. Our study presents an initial characterization of the accessory AMR genes and plasmid replicon types of STEC, which will form the basis of future epidemiological investigations. More work is needed to develop and model epidemiological dynamics at an unparalleled resolution that integrates variation in the core genome, accessory genome, and mobile genetic elements. This is particularly important when investigating pathogen microevolution and disease transmission over very short timescales (e.g., weeks or months).

We recognize the limitations of our study. First, pertinent clinical and epidemiological data associated with each isolate are lacking. We do not have information about the social relationships, if any, of the patients within each putative genetic cluster (e.g., same household, travel history, food consumption history, contact with animals) as these were not provided by healthcare providers to us. Moreover, the 10-SNP threshold we used serves only a proxy for cluster estimation; therefore, accurate identification of disease transmission routes, common sources of infection, or divergence from a common ancestor remains unclear. We are also unable to make inferences as to whether individual serotypes, virulence genes, and AMR gene content are associated with patient outcomes and secondary complications. Second, the surveillance program relies on the receipt of bacterial samples submitted by healthcare providers. Hence, certain regions within the state may be overrepresented, while others remain under-sampled. In our study, there were many more isolates received from the counties Kings, Monroe, Nassau, New York, Suffolk, and Westchester than others. Counties in large cities certainly have a higher number of people than in other parts of New York State that are more rural; consequently, more individuals were surveyed in urban areas. According to the 2020 state census, the population of New York City and its suburbs is approximately 14.1 million, compared with 6.07 million in the rest of the state. Third, we only have genome sequences from human infections. Genomic comparison with STEC derived from ruminants and food products would have helped in uncovering sources of infection and transmission, especially for the 28 core SNP-based genetic clusters we identified. Fourth, *in silico* identification of STs, serotypes, AMR and virulence genes, and plasmid replicon types is highly reliant on the quality of the query sequences as well as the composition of the databases which were interrogated. While we ensured that only high-quality genome sequences are included by using stringent thresholds, we acknowledge that short-read sequences are fragmented and incomplete. Long-read sequences will certainly improve our analyses, especially in reconstructing plasmid sequences and their cargo genes. Notwithstanding these limitations, our study presents an important starting point in understanding the diversity of clinical STEC and their genomic features, thus informing public health and surveillance efforts and food safety measures. These include follow-up validation of transmission events identified using the core gene SNPs, genotype–phenotype correspondence analysis of AMR, and additional sampling in underrepresented counties in New York State.

In summary, we show that serotypes O103:H2 and O157:H7 remain the major causes of STEC infections in New York State, but the rising incidence of less common serotypes is a cause of concern, especially given the large pool of AMR, virulence genes, and plasmids they carry. Genomic surveillance of STEC is critical in enabling targeted, effective, and timely public health interventions.

## Data Availability

The data set supporting the conclusions of this article is included within the article and its supplemental material. Genome sequence data of STEC isolates are publicly available in the NCBI Sequence Read Archive. BioProject and BioSample accession numbers for each genome are listed in Table S1.

## SUPPLEMENTAL MATERIAL

Supplemental figures spectrum.00956-25-s0001.pdf Figures S1 to S3.

Supplemental tables spectrum.00956-25-s0002.xlsx Tables S1 to S7.

ASM does not own the copyrights to Supplemental Material that may be linked to, or accessed through, an article. The authors have granted ASM a non-exclusive, world-wide license to publish the Supplemental Material files. Please contact the corresponding author directly for reuse.
