# Supplementary material for: Genomic epidemiology reveals statewide dispersal of clinical Shiga toxin-producing Escherichia coli and their antimicrobial resistome
Source: Microbiol Spectr. 2025 Nov 14;14(1):e00956-25. doi: 10.1128/spectrum.00956-25 (PMC12772303; doi:10.1128/spectrum.00956-25)
Supplement: Supplemental figures — Figures S1 to S3. [file spectrum.00956-25-s0001.pdf]

Genomic epidemiology reveals statewide dispersal of clinical Shiga toxin-producing *Escherichia coli* and their antimicrobial resistome

Ana Beatriz Garcez Buiatte, Maitiú Marmion, Samara T. Choudhury, Letícia Roberta Martins Costa, Odion O. Ikhimiukor, Samantha E. Wirth, Kimberlee A. Musser, Lisa A. Mingle, Cheryl P. Andam

Supplementary information

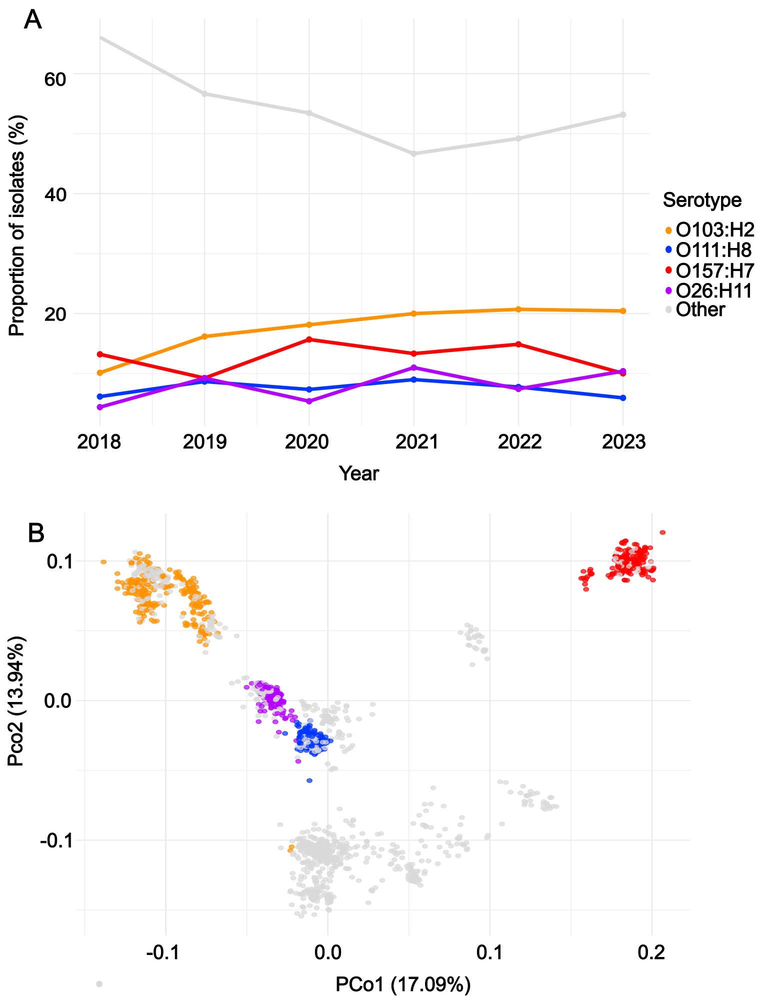

Supplementary Figure S1. (A) Frequency of STEC in New York State over time classified according to serotypes. For visual clarity, the four major serotypes are shown in colored lines and less common serotypes are grouped together in the category "Other". (B) Principal Coordinates Analysis (PCoA) based on Jaccard distances of gene presence/absence across isolates. Points are colored according to serotype.

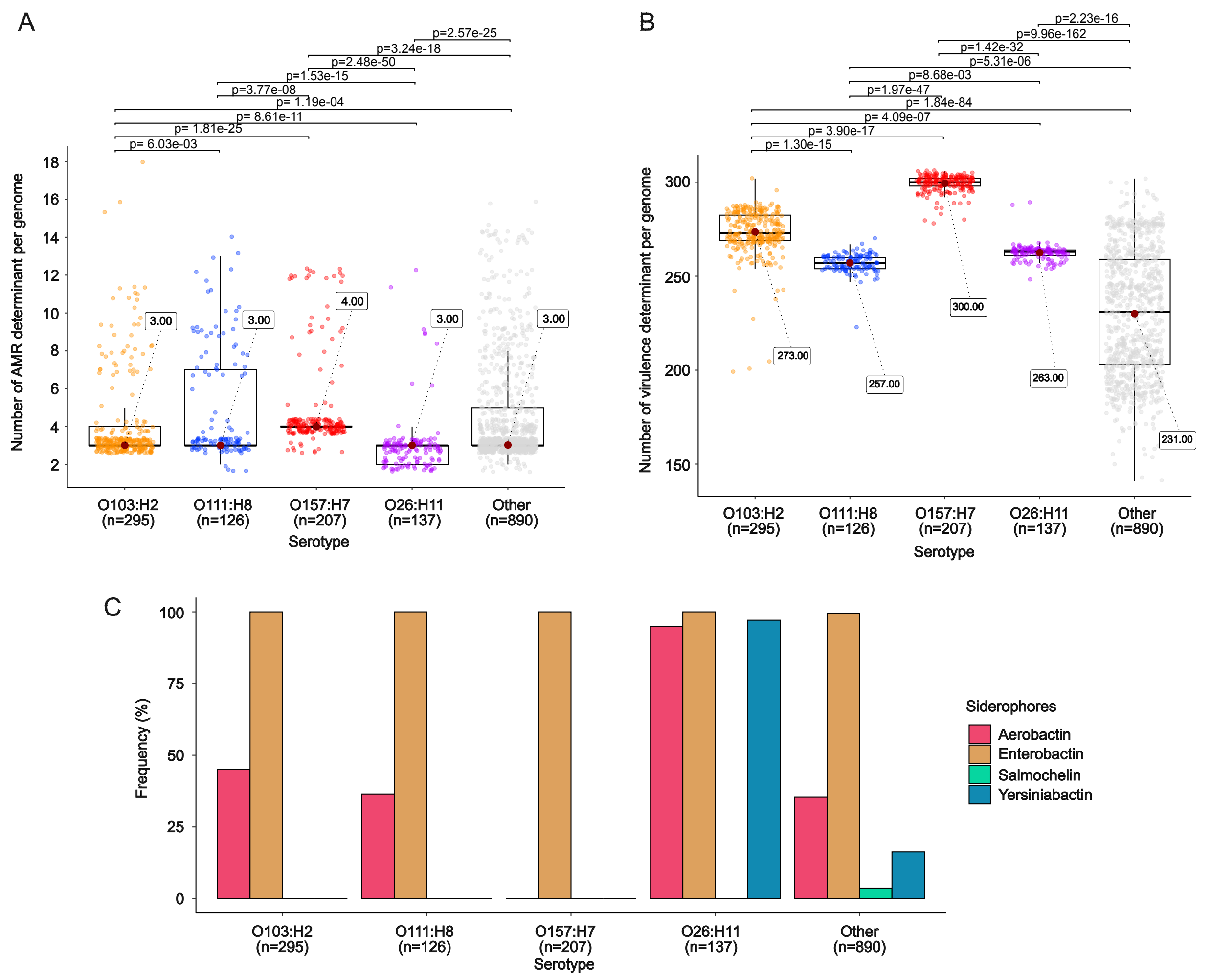

Supplementary Figure S2. Comparison of the number of (A) antimicrobial resistance (AMR) determinants and (B) virulence genes per genome according to serotype. For panels (A) and (B), Kruskal–Wallis tests with Holm–Bonferroni–adjusted *p*-values were used to compare groups. Colored dots represent genomes. The mean value is represented by the red dot, the box represents the interquartile range, the horizontal line in the middle of the box represents the median and the lower and upper ends of the violin jitter plots represent the lowest data point without the outliers and the highest data point without outliers, respectively. (C) Frequency of siderophores across serotypes. For visual clarity, the four major serotypes are shown in colored lines and less common serotypes are grouped together in the category "Other".

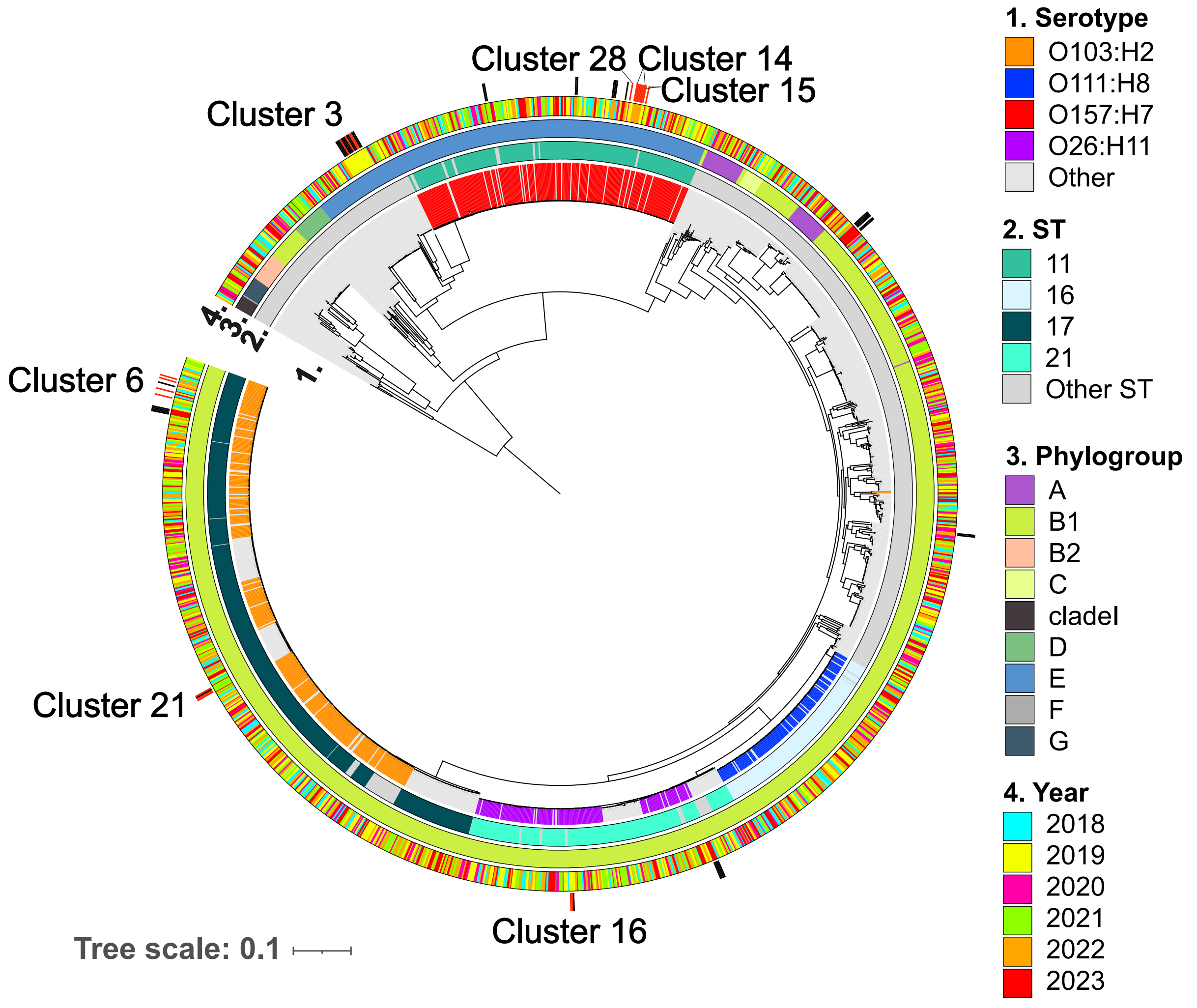

Supplementary Figure S3. Phylogenetic tree showing the genomes associated with disease outbreaks identified in the CDC PulseNet database but not in our study (black bars in the outermost ring), and genomes associated with outbreaks by both PulseNet and our study (red bars in the outermost ring). This phylogenetic tree is identical to that in Figure 1A. The genetic clusters we delineated are based on the 10-SNP threshold in the core genome alignment.
